# A decade of review in global regulation and research of artificial intelligence medical devices (2015–2025)

**DOI:** 10.3389/fmed.2025.1630408

**Published:** 2025-07-17

**Authors:** Siwen Zhang, Yujun Li, Wenxi Liu, Qi Chu, Shasha Wang, Jinyu Li, Yuwen Chen

**Affiliations:** ^1^School of Medical Device, Shenyang Pharmaceutical University, Shenyang, China; ^2^School of Pharmacy, Shenyang Pharmaceutical University, Shenyang, China; ^3^School of Pharmaceutical Engineering, Shenyang Pharmaceutical University, Shenyang, China; ^4^Department of Clinical Laboratory, Peking Union Medical College Hospital, Chinese Academy of Medical Sciences, Beijing, China; ^5^School of Traditional Chinese Materia Medica, Shenyang Pharmaceutical University, Shenyang, China; ^6^School of Business Administration, Shenyang Pharmaceutical University, Shenyang, China

**Keywords:** artificial intelligence medical device, regulatory policy, total product life cycle (TPLC) management, international harmonization, innovation and safety

## Abstract

Artificial intelligence (AI) and medical devices are increasingly integrated, reshaping global diagnostic paradigms. However, the adaptive learning and opaque nature of AI technologies pose significant challenges to traditional regulatory frameworks. In response, regulatory bodies worldwide, including the U.S., EU, China, Japan, and South Korea, have initiated various policies to address the unique risks posed by AI medical devices (AIMD). These efforts aim to balance innovation with patient safety, yet gaps remain in harmonizing standards across regions and ensuring comprehensive oversight. This study provides a comprehensive analysis of the regulatory policies for AIMD from 2015 to 2025 across key global regions. We examine the evolution of these policies, the academic research progress, the limitations of existing regulations, and emerging trends. By reviewing relevant legislation and literature, this paper offers valuable insights for researchers, manufacturers, and regulators to foster the development of robust regulatory frameworks for AIMD.

## 1 Introduction

Since 2015, the integration of artificial intelligence (AI) technology into the healthcare sector has deepened significantly, with AI-based medical devices (AIMD) emerging as a pivotal category in innovative medical practices (1). AIMD leverage machine learning algorithms to analyze multimodal medical data, enabling applications such as real-time sepsis prediction (2), automated retinopathy screening (3), and cancer risk stratification (4). While these advancements hold promise for reducing diagnostic errors and assisting clinicians, their adaptive learning capabilities and algorithmic opacity pose substantial challenges to traditional medical device regulatory frameworks (5).

Regulatory agencies worldwide face the dual challenge of ensuring patient safety while fostering innovation. The U.S. Food and Drug Administration (FDA) pioneered a lifecycle management framework for AIMD through its Digital Health Innovation Action Plan (2017) (6), while the European Union’s Artificial Intelligence Act (AI Act) introduced risk-based classification requirements for AIMD (7). In Asia, regulatory approaches have exhibited regional variations. For instance, China’s National Medical Products Administration (NMPA) issued the Technical Review Guidelines for AIMD (2022) (8), and the Japan Pharmaceuticals and Medical Devices Agency (PMDA) developed an Adaptive AI Regulatory Framework (9), reflecting a balance between algorithmic accountability and regulatory flexibility. Nevertheless, critical gaps persist in AIMD regulatory research. Notably, fewer than 30% of AIMD disclose demographic diversity in training datasets (10), raising concerns about algorithmic bias. Additionally, divergent regulatory standards across regions complicate multinational approvals, while approximately 43% of FDA-approved or recognized AIMDs lack clinical validation data, with only 28% having undergone prospective testing for device validation (11). Although countries have begun establishing regulatory frameworks, existing studies predominantly focus on single regions or technical aspects, lacking a systematic, cross-national, and interdisciplinary synthesis. This hinders a comprehensive understanding of the co-evolution between regulatory developments and academic research, leaving a gap in global perspectives on AIMD regulation.

**Figure 1:**
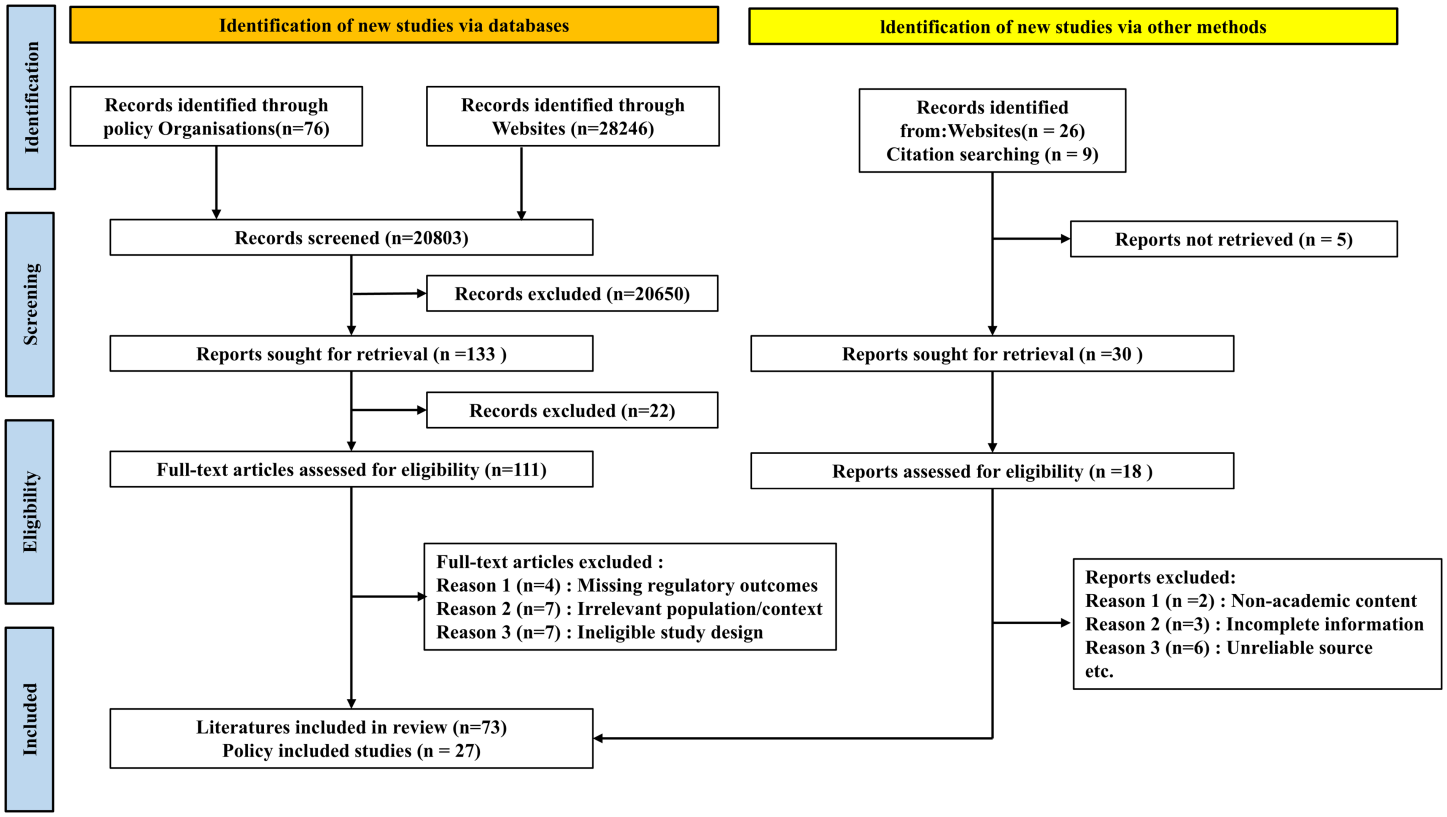
PRISMA flow diagram of literature retrieval process.

This study presents the first comprehensive analysis of AIMD regulatory policies and academic literature across five leading regions—the U.S., EU, China, Japan, and South Korea—from 2015 to 2025. The process of literature retrieval is illustrated in Figure 1. It systematically examines the evolution of regulatory policies, academic advancements, current limitations, and future trends in AIMD governance. By synthesizing regulatory documents and scholarly publications, this work aims to provide researchers, manufacturers, and policymakers with a holistic reference to support the responsible development of AIMD regulations.

## 2 Method

### 2.1 Selection of jurisdictions

This study focuses on the United States, the European Union, China, Japan, and South Korea. These five regions were selected as they represent the world’s largest medical device markets and have been at the forefront of developing pioneering and influential regulatory frameworks for AIMD. Their policies often serve as international benchmarks. While other countries are also developing regulations, a comprehensive review of all global jurisdictions is beyond the scope of a single study. By concentrating on these key regions, this paper provides a deep and comparative analysis of the most developed and globally significant regulatory models.

### 2.2 Search strategy and selection of regulatory policies

A systematic search was conducted to identify official regulatory documents, guidelines, and policy announcements related to AIMD from 2015 to 2025. The search included the official public-facing websites and databases of the respective regulatory agencies: the U.S. Food and Drug Administration (FDA), the European Commission and the European Medicines Agency (EMA), China’s National Medical Products Administration (NMPA), Japan’s Pharmaceuticals and Medical Devices Agency (PMDA), and South Korea’s Ministry of Food and Drug Safety (MFDS). The search was performed using English keywords such as “artificial intelligence medical device,” “AI/ML software,” “software as a medical device (SaMD),” “digital health regulation,” and specific policy names (e.g., “Predetermined Change Control Plan,” “AI Act”). For non-English websites, searches were also conducted using translated native-language equivalents. Documents were included if they were official, final, or draft guidance, regulations, or white papers issued by the competent authority.

### 2.3 Search strategy and selection of academic literature

To review academic research, a systematic literature search was conducted in the PubMed and Google Scholar databases, covering the period from January 2015 to December 2024. The search strategy used a combination of keywords, including: “artificial intelligence medical device,” “AI medical software,” “regulatory science,” “medical device regulation.”

The literature retrieval and screening process is illustrated in the PRISMA flow diagram (Figure 1). For inclusion, articles had to be peer-reviewed and focus on the regulation, policy, ethics, or clinical evaluation of AIMD in one or more of the selected jurisdictions. Articles were excluded if they (1) focused exclusively on the technical development of AI algorithms without discussing regulatory or ethical implications; (2) were not published in English or Chinese; or (3) were editorials, news reports, or opinion pieces lacking substantive analysis. Two authors independently screened the titles and abstracts. Any disagreements regarding inclusion were resolved through discussion with a third author to reach a consensus.

## 3 Global regulatory policy framework

### 3.1 Regulatory policies in U.S.

The U.S. Food and Drug Administration (FDA) employs a risk-based classification system to regulate medical device software, establishing a three-tiered regulatory framework centered on device attribute determination, risk stratification, and approval pathway selection. In 2017, the FDA advanced regulatory modernization through the Digital Health Innovation Action Plan, restructuring its approach to digital health products to ensure timely public access to high-quality, safe, and effective technologies while fostering innovation. This initiative launched pilot programs such as the Software Pre-Certification Program (Pre-Cert Program), which developers demonstrating organizational excellence to accelerate product registration reviews (12). For AIMD, the FDA proposed a Total Product Life Cycle (TPLC)-oriented regulatory model in the 2019 discussion paper Proposed Regulatory Framework for Modifications to AI/ML-Driven Software as a Medical Device, with the core being Predetermined Change Control Plans (PCCP) (13). This framework allows manufacturers to pre-specify algorithm update parameters during premarket submission, enabling post-approval modifications within approved safety boundaries. The 2021 AI/ML Software Action Plan formalized this framework by drafting PCCP-related guidelines and emphasizing post-market performance monitoring (14). The subsequent PCCP Marketing Submission Guidance draft (2023) clarified permissible conditions for algorithmic modifications, further specifying requirements for submitting algorithm change control plans in AIMD marketing applications and delineating modifications exempt from additional approvals (15). In December 2024, the FDA finalized the Guidance on Predetermined Change Control Plans for AI-Enabled Medical Device Software, expanding the scope from machine learning-based devices to all AI-driven technologies compared to earlier drafts (16). This final guidance provides standardized recommendations for AIMD manufacturers on implementing predefined change control processes.

In addition to its core regulatory initiatives, the FDA has pursued complementary strategies to modernize oversight. In 2020, the agency established the Digital Health Center of Excellence to centralize governance of digital health technologies, including AI-driven medical devices. Concurrently, it finalized guidance for Clinical Decision Support Software (CDS), reclassifying specific clinician-facing AI-based CDS with interpretative functionality as non-medical devices—a regulatory refinement intended to alleviate burdens for low-risk AI applications while preserving oversight of higher-risk clinical tools (17). In 2021, the FDA collaborated with international regulatory bodies to endorse the Good Machine Learning Practice (GMLP) principles, articulating 10 foundational criteria spanning data integrity, model transparency, independent validation, and continuous performance monitoring. These guidelines aim to standardize AIMD development practices, ensuring safety, effectiveness, and reproducibility across the AI/ML product lifecycle (18). In 2025, the FDA issued draft guidance that includes recommendations to support development and marketing of safe and effective AI-enabled devices throughout the device’s Total Product Life Cycle (19).

Notably, while regulatory frameworks have evolved, the FDA has authorized a substantial number of AIMDs through existing mechanisms. As of October 2024, the agency has granted 1,016 AIMD authorizations, with approvals demonstrating exponential growth in recent years—from 6 in 2015 to 113 in 2021 and surging to 223 in 2023 (20). The majority of approved products are radiology-focused, obtained via the 510(k) pathway, while a smaller subset of innovative technologies utilize the *De Novo* or PMA pathways. Notably, breakthrough AI/ML devices have also been incorporated into the Breakthrough Devices Program, accelerating review timelines. Collectively, U.S. regulatory policies are characterized by the flexible application of guidelines and pilot programs within existing legal frameworks to guide the safe and effective commercialization of AIMD (21). With the implementation of policies such as PCCP, the FDA is poised to further refine software update management for AIMD, achieving a balance between fostering innovation and ensuring patient safety.

A Comparative overview of AIMDs Regulation in the USA, Europe, China, Japan and South Korea is shown in Table 1.

**Table 1:** A comparative overview of AIMDs regulation in the USA, Europe, China, Japan and South Korea.

Country/ Items	USA	Europe	China	Japan	South Korea
Regulatory agencies	FDA (U.S. Food and Drug Administration)	CE (Conformité Européenne)	NMPA (National Medical Products Administration)	PMDA (Pharmaceuticals and Medical Devices Agency)	MFDS (Ministry of Food and Drug Safety)
Core regulations	Federal Food, Drug, and Cosmetic Act (FD&C Act)	Medical Device Regulation (MDR 2017/745); Artificial Intelligence Act	Regulations on the Supervision and Administration of Medical Devices	Pharmaceutical and Medical Device Act (PMD Act)	Medical Device Act
Approval pathways	1. PMA (Premarket Approval): High-risk devices (Class III) 2. 510(k): Moderate/low-risk devices (Class II) 3. *De Novo*: Novel devices (Class I/II)	1. Class I: Self-certification 2. Class IIa/IIb/III: Notified Body review 3. IVDs: Risk-based classification	1. Class I: Filing system 2. Class II/III: NMPA approval (clinical trials required)	1. Class I: Filing system 2. Class II-IV: PMDA or third-party certification	1. Class I: Self-assessment 2. Class II-IV: MFDS/designated agency approval (KC certification)
AI-specific guidelines	1. Digital Health Innovation Action Plan (Pre-Cert Pilot Program) 2. AI/ML-Based Software as a Medical Device (SaMD) Framework 3. Guidance on Marketing Submission Recommendations for Predetermined Change Control Plan for Artificial Intelligence-Enabled Device Software Functions (PCCP)	1. MDR Annex VI (software requirements) 2. MDCG Guidance on SaMD 3. Artificial Intelligence Act (AI Act)	1. Guidance principles for the classification and definition of AIMD products 2. Registration Review Guidelines for AIMDs and Algorithm Performance Evaluation Methods for AIMD and Algorithm Performance Evaluation Methods for AI Medical Devices	1. Review Issues and Recommendations on AI Medical Diagnostic Systems and Medical Devices 2. Review Issues and Recommendations on AI Medical Diagnostic Systems and Medical Devices Pharmaceutical and Medical Device Act (PMD Act) 3. DASH for SaMD Accelerated Review Reform Program 4. Guidance on Application Risk Assessment for Generative AI in Medical Devices	1. KC Safety Certification 2. Guidance on the Review and Approval of Artificial Intelligence(AI)-based Medical Devices 3. Safety Evaluation Guidelines for Generative AI Medical Software 4. Artificial Intelligence Basic Law
Regulatory features	1. Risk-based classification 2. Emphasis on algorithm transparency 3. Pre-Cert programs	1. Unified MDR/IVDR framework 2. Strict clinical evidence requirements 3. Algorithm updates require re-evaluation	1. Localized clinical trials 2. Data security compliance	1. Phased approval (technical and clinical review) 2. Algorithm interpretability requirements	1. KC certification 2. Local data requirements 3. Cybersecurity compliance

### 3.2 Regulatory framework in European Union

The European Union (EU) enacted the revised Medical Device Regulation (MDR) in 2017, with full implementation effective May 2021. This regulation substantially strengthens oversight of standalone software, replacing the more permissive framework under the Medical Devices Directive (MDD). Central to these changes is Annex VIII, Rule 11, which redefines software classification criteria (22): systems providing diagnostic or therapeutic decision-making information are classified as Class IIa, while applications involving life-threatening clinical decisions automatically qualify as Class III. Systems that may induce serious clinical deterioration are typically designated as Class IIb devices, and physiology monitoring tools typically fall under Class IIa, with only non-diagnostic tools retaining Class I status. These provisions have resulted in the majority of AI-driven medical software being reclassified as high-risk. Manufacturers must now establish MDR-compliant quality management systems (QMS), conduct rigorous clinical evaluations and performance validation, and obtain Conformité Européenne (CE) marking before marketing AI products in the EU.

The EU has consistently advanced the development of Trustworthy AI. Since 2019, it has released key policy documents including the Ethics Guidelines for Trustworthy AI, Policy and Investment Recommendations for Trustworthy AI, and White Paper on Artificial Intelligence: A European Approach to Excellence and Trust, emphasizing that AI systems must fulfill three core requirements throughout their lifecycle: lawfulness, ethical alignment, and robustness (23–25). Furthermore, the EU has prioritized general AI regulation. In 2021, the European Commission proposed the Artificial Intelligence Act (AI Act), the world’s first legally binding framework for AI (7). The Act designates healthcare AI systems as high-risk AI, mandating compliance with transparency, risk management, and data governance standards while establishing a comprehensive foundation for sustainable AI deployment (26). However, experts caution that the AI Act may inadequately address the unique regulatory conditions of medical AI under the Medical Device Regulation (MDR), necessitating harmonization between the two frameworks to avoid disproportionate compliance burdens (27).

Overall, the EU has comprehensively integrated AIMD into stringent regulatory frameworks through the MDR, raising entry barriers for market access. While enhancing safety assurances, this framework has sparked concerns about potential stifling of innovation. In response to these challenges and widespread industry concerns, EU policymakers are taking steps to ease the regulatory burden. Notably, a European Parliament resolution in early 2024 called for amendments to the MDR to improve its implementation, address bottlenecks, and support the availability of medical devices, signaling a move toward a more pragmatic balance between safety and innovation (28).

Furthermore, to harmonize the assessment process, European Notified Bodies have developed joint guidance, such as the Joint paper from the Notified Bodies on AI in medical devices, which outlines a standardized questionnaire for the review of AIMD, representing a key practical step in regulatory assessment for market access (29).

### 3.3 Regulatory framework in China

The NMPA of China has systematically established a regulatory framework for AIMD through a series of normative documents. In August 2015, the NMPA issued the Technical Review Guidelines for the Registration of Medical Device Software, which first outlined requirements for medical device software—including functional categorization, validation testing, and clinical evaluation—and mandated that software employing AI algorithms for diagnostic or detection purposes must submit clinical trial data to substantiate efficacy (30). To address emerging challenges posed by advancements in deep learning technologies (a core algorithmic in AI), particularly in medical imaging, regulatory authorities have dynamically updated their policies to ensure compliance and safety. In July 2019, the NMPA issued the Review Key Points for Deep Learning-Assisted Decision-Making Medical Device Software, a guideline specifically addressing AI algorithm-based medical software (31). This document outlines critical evaluation criteria, including requirements for training data quality, validation methods for algorithm performance, clinical trial design, and safety risk mitigation. A cornerstone of the regulatory framework lies in clearly defining the management attributes and classification criteria for AIMD. In July 2021, the NMPA issued the Guidelines for Classification and Definition of AI Medical Software, which formally defined AIMD as “medical devices that utilize artificial intelligence technology to achieve medical purposes based on medical device data” (32). These guidelines further clarified the regulatory classification of AIMD, establishing pathways for product registration. The NMPA classifies AI-driven auxiliary diagnostic or detection software as Class III high-risk medical devices, while a small subset of products are designated as Class II medium-risk devices. Generally, AIMD are not categorized as Class I, which means low-risk devices, aligning with their inherent risk profiles and the risk classification framework of the IMDRF (33). Building on the clarified classification framework, the NMPA issued the Technical Review Guidelines for AIMD in March 2022, a landmark policy document systematically outlining technical requirements for AIMD development and registration. The guidance emphasizes whole life cycle quality control for AI, mandating rigorous training data representativeness and validation while requiring developers to submit detailed documentation—including source code summaries, algorithm efficacy proofs, and performance validation reports (34). Clinical evaluation through rigorously designed trials is mandated to demonstrate safety and effectiveness in real-world clinical settings. For self-learning algorithms, developers must explicitly define learning mechanisms and scope, while technical measures must be implemented to “lock” the algorithm post-market, prohibiting automatic updates unless re-submission for modification is approved. This guideline establishes quality and safety benchmarks for AIMD evaluation in China, aligning with global regulatory expectations while addressing the unique challenges posed by adaptive AI technologies (8).

Beyond the aforementioned regulatory documents, China has further advanced AIMD oversight through standardization and institutional innovation. In July 2019, the NMPA launched the AIMD Innovation Collaboration Platform, convening 14 medical institutions, research organizations, and regulatory authorities to address challenges posed by rapid AI technological evolution. In recent years, China has promulgated multiple industry standards, including the Guidance on Algorithm Performance Evaluation Methodology for AIMD, and specialized guidelines such as the Guidelines for Registration Review of AIMD for Pulmonary Nodule Detection in CT Images (35). These regulatory refinements reflect China’s systematic approach to balancing innovation acceleration with safety assurance. In 2023, a consortium led by Zhejiang University in collaboration with the National Institutes for Food and Drug Control and other authoritative institutions published the Expert Consensus on General Methods for Performance Evaluation of Artificial Intelligence Medical Devices. This landmark document systematically consolidates standardized testing methodologies for AIMD (36). To encourage innovation, China has opened its Special Approval Channel for AIMD, enabling 47 AIMD products to enter this accelerated pathway since 2018, with an average review cycle shortened by 83 days, while progressively implementing real-world data pilot programs to explore methods for leveraging RWD in regulatory decision-making. These policy refinements have yielded measurable progress: as of December 2024, the NMPA has approved 126 Class III AIMDs, predominantly in high-impact domains such as pulmonary nodule detection, intracranial hemorrhage identification, and electrocardiogram analysis, with most products delivered as standalone software (37). China has significantly accelerated AIMD review timelines in recent years, a direct outcome of timely regulatory guidance such as the Quality Requirements and Evaluation for Artificial Intelligence Medical Devices and specialized protocols like those for pulmonary nodule detection in CT imaging (38). By issuing targeted guidelines that clarify compliance benchmarks—such as algorithm training data quality, validation methodologies, and clinical trial evidence requirements—regulators have standardized industry R&D practices, improved submission quality, and expedited approvals, thereby aligning China’s regulatory framework with global standards while addressing the unique challenges of adaptive AI.

### 3.4 Regulatory framework in Japan

Japan’s regulatory framework for standalone software as medical devices was established relatively later than other jurisdictions (39). The amended Pharmaceuticals and Medical Devices Act (PMD Act) of 2014 marked a pivotal transition by formally recognizing software as a distinct category of medical devices, thereby incorporating standalone software into Japan’s medical device regulatory system (40). In December 2017, the PMDA established AI Subcommittee released the Review Issues and Recommendations on AI Medical Diagnostic Systems and Medical Devices (41). This report provides a comprehensive analysis of the unique characteristics and risks inherent to AIMD, outlining key considerations for review and deployment, including AI model continuous learning capabilities, result interpretability, and training data quality. It establishes the landmark conceptual framework for AIMD regulation of PMDA, which has profoundly influenced subsequent regulatory guidance development (42). Building on this foundation, Japan’s NIHS released the Report on the Artificial Intelligence Review Working Group in March 2019 as a draft guideline for AIMD review, laying the groundwork for formal regulatory standards. The report systematically identified barriers to AIMD development across technical phases and proposed nine stage-specific challenges and solutions during the subsequent Accelerated Medical AI Development Consortium meeting. These included ethical review frameworks, data labeling standards, clinical validation methodologies, and streamlined review processes (40). This foundational research provided critical evidence for Japan’s AI regulatory strategy, culminating in significant policy advancements such as regulatory revisions to enhance adaptability for emerging AI technologies. In November 2019, Japan enacted the revised PMD Act, introducing novel regulatory mechanisms such as conditional early approval and the Post-Approval Change Management Protocol (PACMP). Under the PACMP framework, companies may submit proposed product modification plans during initial submissions; once approved by the PMDA, subsequent changes within the approved scope may undergo streamlined review (43). This mechanism, analogous to the FDA’s PCCP, addresses regulatory challenges posed by AI algorithm’ continuous learning capabilities. Japan pioneered the codification of such adaptive frameworks into law, establishing an innovative regulatory approach for post-market updates of AIMD.

In December 2018, Japan approved its first AIMD—an AI software designed to assist in distinguishing intestinal tumor lesions using endoscopic imaging—after rigorous clinical trial data requirements by the PMDA to demonstrate tumor detection sensitivity and specificity (44). While subsequent AIMD approvals in Japan have remained limited, most have been classified as Class III high-risk devices. The PMDA maintains a cautious approach for each AIMD, mandating clinical validation within Japanese patient populations and ensuring physicians fully comprehend AI-generated outputs. To address the surge in digital health products, the MHLW introduced the DASH for SaMD initiative in March 2020, proposing measures such as streamlined innovative software review processes, integration of external expert participation in AIMD evaluations, and enhanced pre-submission communications with developers to improve regulatory efficiency (45). By March 2021, Japan began exploring the inclusion of AIMD in its national health insurance reimbursement system, addressing economic evaluations and reimbursement policies for AI-driven medical technologies (46).

In summary, Japan has established a comprehensive policy framework for AIMD that integrates research discussions, regulatory revisions, guidelines development, and accelerated reviews. Despite its later entry into this field, Japan has emerged as one of the few countries with a mature regulatory system for AIMD. The emphasis of Japan’s policies lies in balancing flexibility with safety assurances. This dual focus reflects both the cautious approach of Japanese regulators to AI technology risks and their unwavering commitment to fostering medical AI innovation.

### 3.5 Regulatory framework in South Korea

South Korea has demonstrated a responsive and proactive regulatory approach toward AIMD. The Ministry of Food and Drug Safety (MFDS) approved Korea’s first AIMD software in 2017—a tool for electrocardiogram analysis—followed by multiple radiology imaging-assisted AIMD between 2018 and 2019, establishing the country as one of the earliest to deploy AI-driven medical software in clinical settings. In 2023, a total of 64 kinds of AIMDs were approved by MFDS and obtained certifications worldwide, an increase of 17 kinds compared to the previous year. Among them, 9 kinds were imported products (accounting for 14.1%), and 55 kinds were domestic products (accounting for 85.9%) (47).

To address the rise of AIMD, MFDS has established a robust regulatory framework through legislative and guideline-driven approaches. On one hand, specialized legislation was enacted to support the medical AI industry. The Act on Nurturing the Medical Devices Industry and Supporting Innovative Medical Devices, formally implemented in May 2020, empowers the MFDS to identify innovative medical devices and provide priority reviews and comprehensive support across R&D, approval, and post-market phases (48). As of October 2021, 16 products—including 10 AIMDs—had been designated as innovative medical devices under this framework. Additionally, the law incorporates elements of the FDA’s Pre-Cert framework, enabling MFDS to certify developers’ R&D and quality management capabilities, thereby streamlining product-specific review requirements (49). On the other hand, MFDS has prioritized refining detailed AIMD review guidelines to align with evolving regulatory needs. In September 2019, MFDS released the Guideline for Review and Approval for Software Medical Devices, outlining general requirements for AI software (50). By May 2022, MFDS had finalized the Guidelines on the Review and Approval of Artificial Intelligence-based Medical Devices, further refining regulatory standards for AI-driven systems. These documents emphasize MFDS’ dual focus on accelerating innovation and ensuring safety, requiring developers to demonstrate algorithmic transparency, clinical validation robustness, and post-market surveillance capabilities. The guidelines apply to all AI-based medical device software, specifying the technical documentation required for AIMD licensing in South Korea, including clinical trial data or literature evidence validated within the Korean population. They mandate that clinical decision support and computer-aided detection or diagnosis software be regulated as medical devices (51).

South Korea’s regulatory policies are distinguished by their openness to collaboration and alignment with global standards. The MFDS actively participates in the International Medical Device Regulators Forum, joining as a full member in 2017 and assuming the IMDRF chairmanship in 2021 (52). Under its leadership, South Korea spearheaded the development of IMDRF’s Key Terms and Definitions for Machine Learning-enabled Medical Devices (53), while fully adopting the IMDRF’s Good Machine Learning Practices for Machine Learning-enabled Medical Devices (GMLP) into domestic guidelines. This strategic integration ensures South Korea’s regulatory framework remains synchronized with global advancements led by the FDA, EU, and other key stakeholders.

**Figure 2:**
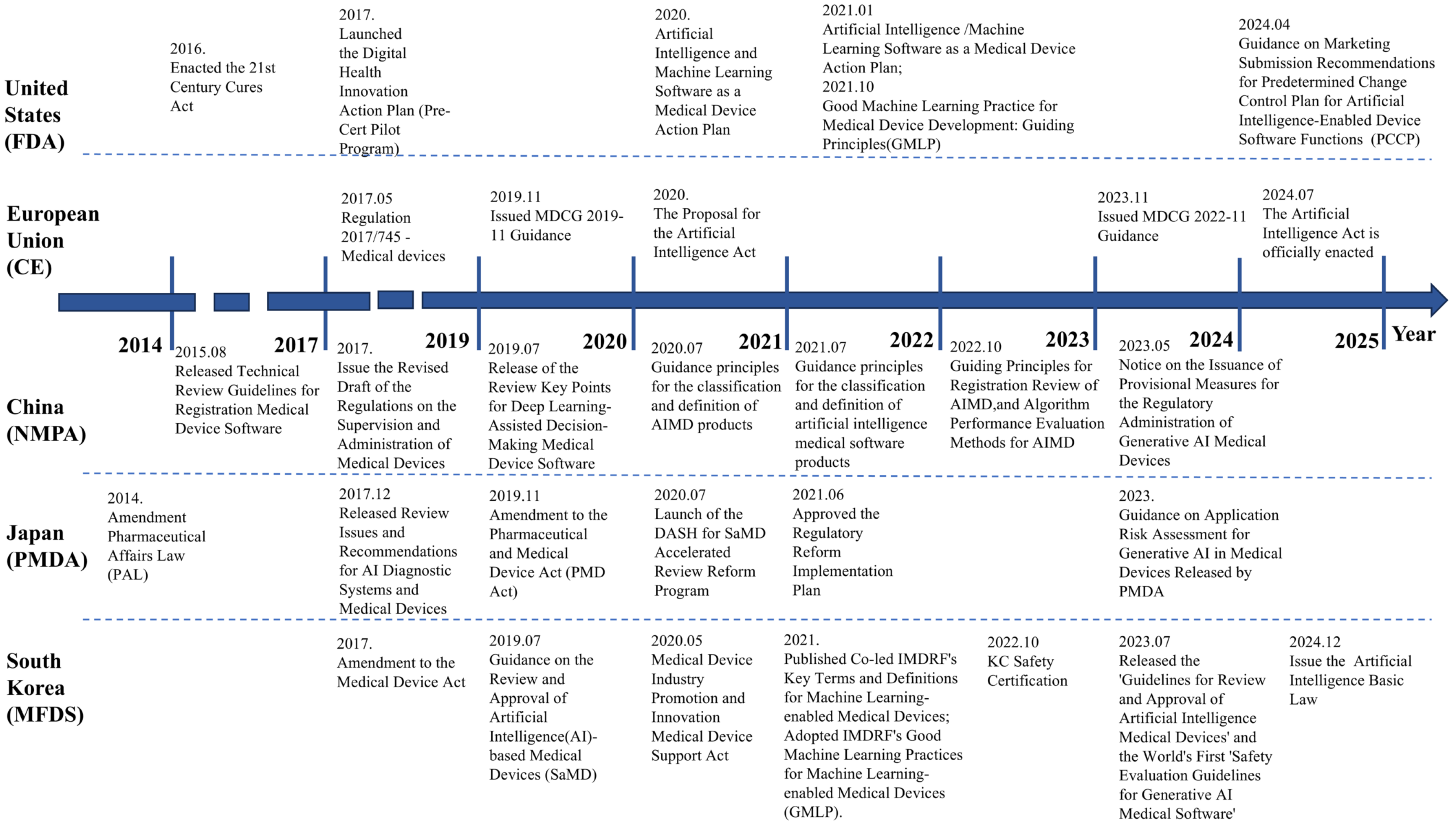
The timeline of AIMD regulation in the U.S., EU, China, Japan, and South Korea.

South Korea also prioritizes emerging trends in AI. In July 2023, it released the world’s first “Safety Evaluation Guidelines for Generative AI Medical Software,” establishing proactive safety assessment frameworks for large medical language models. By combining legal incentives with technical guidelines, South Korea has built a flexible and efficient AIMD regulatory system. Regulatory authorities emphasize advancing global digital health collaboration through bilateral workshops and training programs, enhancing both domestic enterprises’ global competitiveness and South Korea’s influence in international medical device regulation. The timeline of AIMD regulation in U.S., EU, China, Japan, and South Korea is illustrated in Figure 2.

## 4 Academic research overview

**Figure 3:**
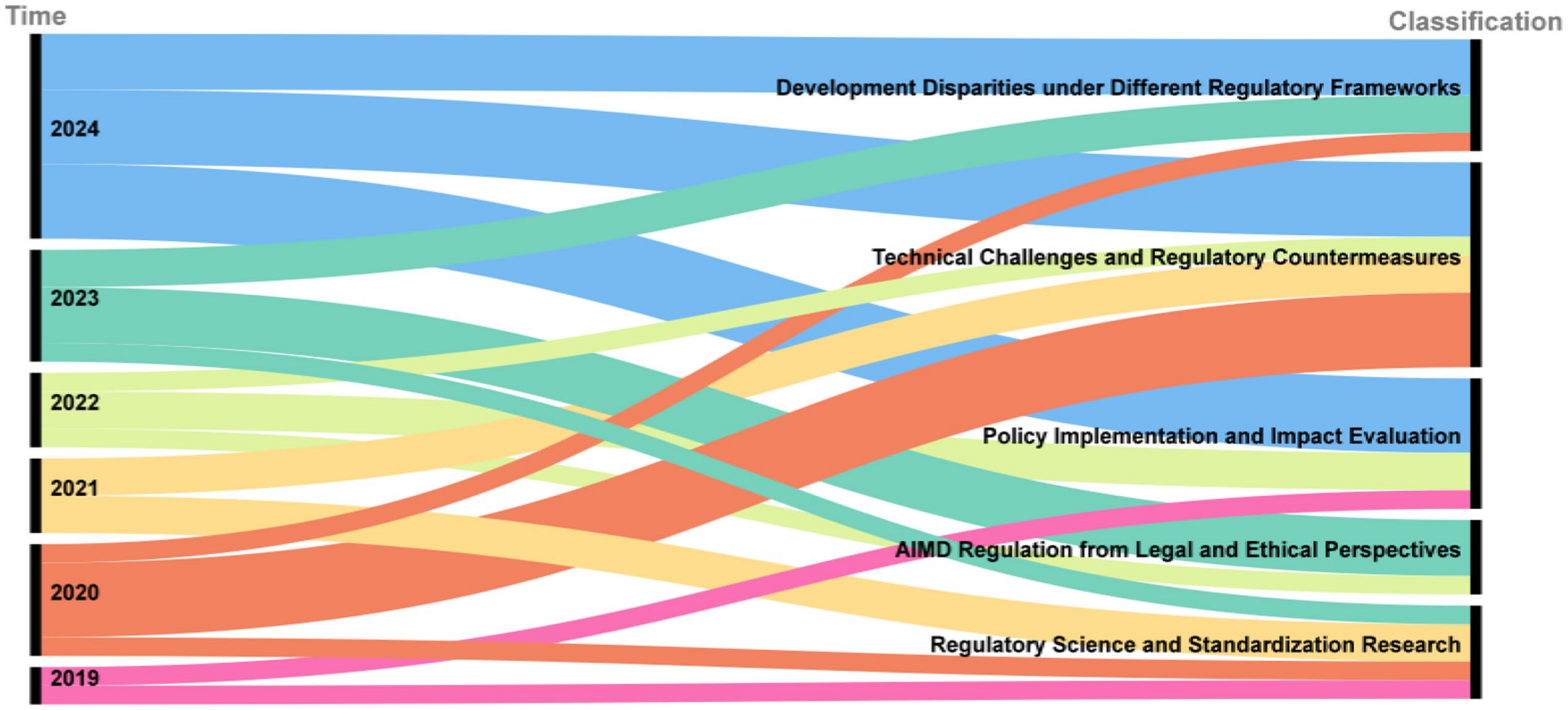
Sankey diagram of academic literature publication years and topics.

**Figure 4:**
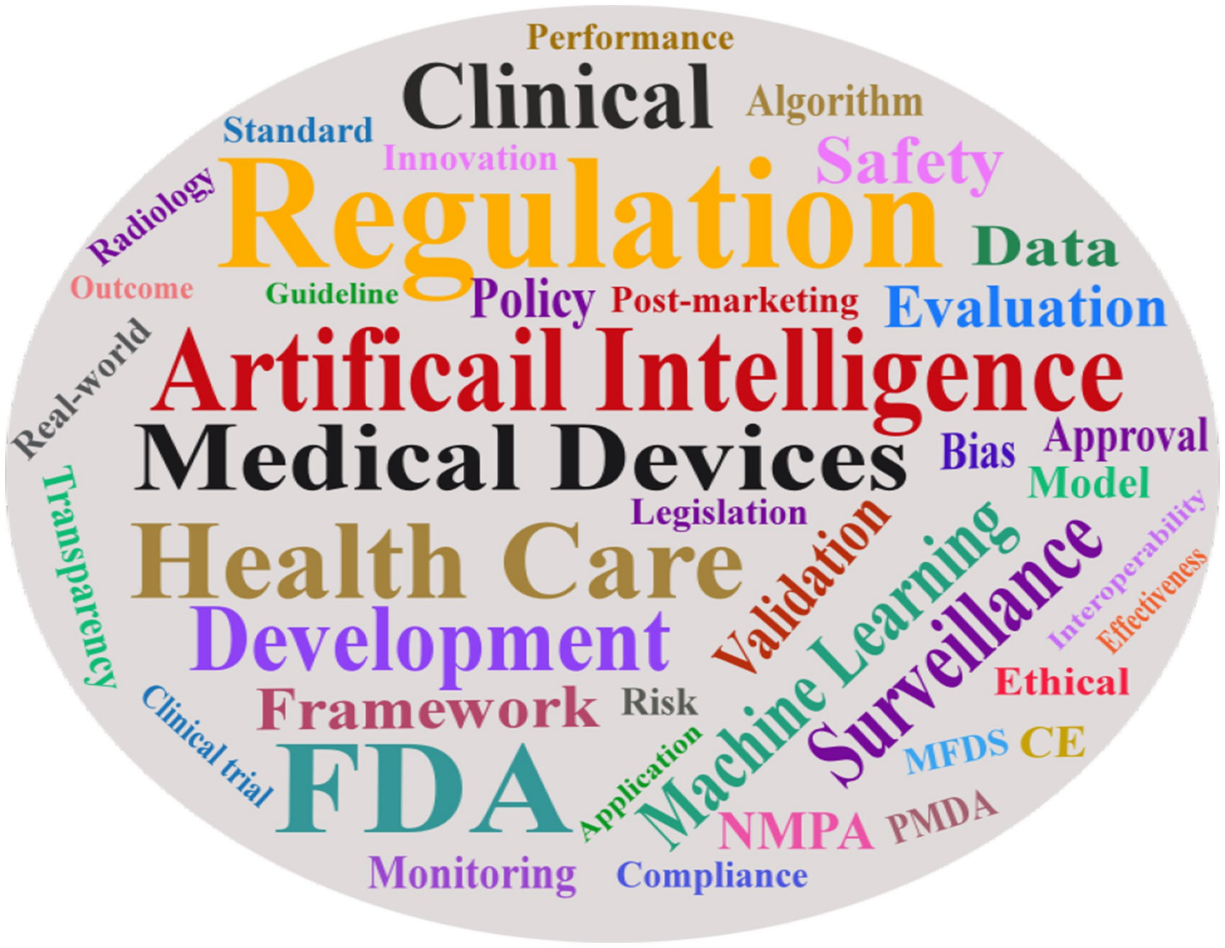
Word cloud diagram of the literature keyword.

In response to the evolving regulation of AIMD, the academic community has conducted extensive research. As illustrated in the Sankey diagram in Figure 3, academic inquiry has shifted over time. Early research (2019–2020) focused on foundational legal, ethical, and standardization issues. This was followed by a wave of studies evaluating policy implementation and its real-world impact (2021–2022). More recently (2023–2024), the literature has concentrated on specific technical challenges, such as data bias and algorithm updates, and on analyzing developmental disparities under different global regulatory frameworks. This section reviews the key themes that have emerged from this body of research, using publications from PubMed and Google Scholar. The word cloud diagram of the literature keywords is presented in Figure 4.

### 4.1 Development differences under different regulatory frameworks

Numerous studies have focused on cross-national disparities in regulatory frameworks, with comparative analyses of policies from major regulatory agencies highlighting significant variations in the classification, definition, and market access requirements for AIMD. A critical analysis of the regulatory frameworks reveals fundamental differences in their core philosophies. Clark et al. contrasted the flexible, guidance-driven approach of the U.S. FDA with the EU’s legally binding Medical Device Regulation (MDR). The U.S. model, which emphasizes process controls and post-market data, fares better for fostering rapid innovation, as evidenced by the 85% of AIMDs approved via the streamlined 510(k) pathway. However, this flexibility comes at the cost of potential risk, as it relies on comparisons to older devices and may overlook the dynamic nature of novel AI algorithms. Approximately 85% of AIMD in the U.S. are approved via the 510(k) fast-track pathway, which relies on historical device comparisons to streamline review but often overlooks the dynamic iterative nature of AI algorithms (54). In stark contrast, the EU’s framework fares better in ensuring upfront patient safety by mandating rigorous pre-market validation and classifying most AIMD as high-risk, which requires extensive clinical evidence before market entry. The drawback to this safety-first approach is a significant increase in compliance costs and certification timelines, which can stifle innovation and has reportedly led some manufacturers to withdraw products from the EU market (7). While this enhances safety, it has resulted in up to 75% of medical devices in some countries being at risk of unavailability (55). This emerging issue has drawn significant attention as manufacturers increasingly withdraw from the EU market due to mounting compliance costs and prolonged certification timelines (56).

Peng et al. analyzed China’s regulatory evolution through a series of guidelines issued by the NMPA since 2015, describing a “stepwise approach” that progresses from foundational regulations to specialized guidance documents, gradually refining the oversight framework (57). While China has aligned with international standards in areas like modification control, gaps remain in ethical review processes for Class III devices, particularly concerning data security oversight (58). These comparative studies underscore the trade-offs inherent in different regulatory models: stringent oversight ensures safety but may impede innovation, whereas lenient policies foster rapid development but require robust risk mitigation (59).

Japan and South Korea, as leading medical technology hubs in East Asia, have adopted distinctive regulatory strategies to balance innovation acceleration with domestic industry support. Japan’s “dynamic approval” system employs risk-tiered management and flexible review criteria, significantly lowering market entry barriers for AIMD. Japan’s AI medical device regulation demonstrates the characteristics of “flexible market access combined with dynamic supervision.” Firstly, both Japan and the United States face challenges with regulatory lag in their AI medical device oversight systems, with both countries spending 3–4 years establishing regulatory frameworks capable of adapting to dynamic algorithms. Regarding algorithm management, Akio Kurokawa noted that Japan requires companies to submit algorithm update plans to address iterative needs, but unlike the U.S. (which mandates specific model retraining cycles), this flexible approach grants companies greater autonomy while simultaneously imposing higher demands on regulators to continuously evaluate algorithmic performance (60, 61). South Korea has adopted a dual-track strategy of “strict regulation and industrial incentives” to promote the development of AIMD. The MFDS has optimized regulations and data policies to support this progress. For high-risk AIMDs, the MFDS mandates the submission of clinical validity data and requires premarket review. Even after approval, products must undergo New Health Technology Assessment to qualify for health insurance reimbursement, a process that can take up to 3 years. Meanwhile, to address the limitations of data privacy regulations on AI, the government permits the use of raw medical data under secure conditions, thereby reducing the compliance risks for enterprises (62, 63). Despite calls by scholars like Warraich et al. for regulatory convergence through platforms such as IMDRF, fundamental conflicts between “process control” and “comprehensive validation” paradigms continue to hinder substantive international cooperation (1, 64).

In summary, these divergent paths highlight a central regulatory trade-off. The U.S. model prioritizes flexibility to accelerate market access, which is arguably better for a rapidly evolving field, but it places a heavier burden on post-market surveillance to catch potential issues. Conversely, the EU model prioritizes pre-market assurance of safety and efficacy, which is better for minimizing initial risk but can slow innovation. Meanwhile, the hybrid models emerging in Japan and South Korea, with adaptive mechanisms like PACMP and innovative device designations, represent pragmatic attempts to capture the benefits of both approaches—a direction that may offer a path forward for future international harmonization.

### 4.2 Policy implementation and impact

Some studies have quantitatively counted the approval status and clinical evidence of AIMD to evaluate the effectiveness of current regulatory policies. For example, Joshi et al. analyzed 691 AIMDs publicly listed by the FDA as of October 2023 and found that they were mainly concentrated in the field of radiological diagnosis (65). They focused on the performance claims and clinical supporting evidence of these approved products, pointing out that most of them only provided data on indicators such as diagnostic accuracy, lacked reports on long-term clinical effectiveness and safety, and there was a risk of a broken chain of evidence. Similarly, Fraser et al. investigated 100 CE-marked AIMD for radiological diagnosis and found that published validation studies primarily emphasized diagnostic accuracy, with minimal assessment of patient outcomes (66). These findings have sparked debate regarding the adequacy of existing regulatory frameworks, suggesting that current clinical validation requirements may not sufficiently capture AI’s broader clinical impact. Khinvasara et al. proposed implementing phased approvals and post-market evaluation mechanisms, allowing products to serve patients while collecting real-world data to refine clinical understanding (67).

Svemp et al. analyzed the impact of the EU MDR on digital health products, observing that limited regulatory resources led to certification delays and market withdrawals among SMEs, highlighting the need for enhanced regulatory capacity (56). In contrast, China’s revised Regulation on the Supervision and Administration of Medical Devices has strengthened post-market surveillance, mandating continuous clinical follow-up data for high-risk AIMD and requiring disclosure of demographic characteristics (e.g., age, gender) (68). However, challenges persist in enforcement, including high compliance costs for manufacturers and inconsistent regional data collection standards.

### 4.3 Technological challenges and solutions

A substantial body of literature has examined the unique technical challenges posed by AIMD and corresponding regulatory strategies. Algorithmic updates and continuous learning represent predominant research themes. Gerke et al. identified a critical paradox wherein 78% of manufacturers suspended algorithm iterations due to re-certification costs, resulting in “frozen AI” systems that contradict the adaptive potential of medical AI (64, 69). In response, emerging studies advocate for “regulated machine learning” frameworks, highlighting the FDA’s PCCP and proposed EU regulations as pioneering approaches (70). These works emphasize the need to quantitatively define “allowable modification ranges” and implement robust post-market surveillance to validate algorithmic improvements (67). Simulation studies suggest that dynamic monitoring mechanisms—such as employing ROC-AUC fluctuation thresholds rather than static performance benchmarks—could enable limited self-training while maintaining safety (71, 72). Concurrently, regulators are urged to require detailed algorithmic update protocols in submissions.

The issue of data bias has garnered significant attention, with Minssen et al. demonstrating that uneven training data distributions can yield divergent performance across demographic groups (17). Alarmingly, only 3.6% of FDA-approved devices reported racial composition data, resulting in a 12.7% higher missed diagnosis rate for African Americans versus Caucasians in diabetes screening algorithms (73, 74). Current regulations in both the US and EU address bias through generic risk management requirements, prompting calls for specific guidelines mandating subgroup performance metrics and bias mitigation strategies during development. Quinn et al. highlight the “black box” nature of deep learning models as undermining clinical trust and complicating accountability—76% of AI diagnostic errors could not be traced to specific algorithmic components, fostering reliance on ambiguous “final decision authority” protocols that may exacerbate malpractice disputes (75, 76). Miguel et al. propose regulatory mandates for human-interpretable outputs, including decision-influencing features and rationale displays (77). The EU AI Act’s transparency requirements for high-risk systems exemplify this approach (78).

### 4.4 Legal and ethical

AIMD regulation also involves discussions on the legal and ethical level. Some legal scholars have analyzed whether current regulations adequately cover the new risks brought by AI. The regulatory landscape for AIMD extends beyond technical considerations to encompass critical legal and ethical challenges. Legal scholars have scrutinized whether current frameworks adequately address novel risks posed by AI in healthcare. Liu et al. highlight that while AIMD enhance diagnostic quality, they simultaneously introduce ethical dilemmas including algorithmic bias, ambiguous liability, privacy breaches, and threats to health equity - necessitating a patient-centric ethical governance framework (74). Van et al. identify persistent age-related biases in AI systems that may exacerbate health disparities among elderly populations. Their analysis reveals that while EU regulations partially address technical biases, contextual biases remain unaddressed, failing to comprehensively mitigate AI-driven health inequalities (79). McKee, M et al. pointed out that the existing regulatory framework still has deficiencies in balancing the contradictions of patient safety and fairness, credibility and effectiveness, clinical boundaries and algorithm updates. It is recommended to reconstruct a risk framework with patient safety as the core, incorporate AI limitations into the MDR intended use statement, and promote the coordination of regulation and clinical value through dynamic guidelines (80). The ambiguity surrounding liability allocation between manufacturers and clinicians remains a contentious issue. Current frameworks lack clarity when diagnostic errors occur due to physician reliance on AI recommendations, creating legal gray areas that undermine public trust (81). Mika et al. advocate for an integrated “ethics audit-shared responsibility-collaborative governance” framework, emphasizing lifecycle ethical assessments, dynamic monitoring, and multi-stakeholder cooperation to balance innovation with risk mitigation (82). Parallel calls exist for specific legislation to delineate liability and prevent accountability gaps from hindering AIMD adoption.

Ethicists emphasize that effective regulation must transcend safety and efficacy to address informed consent, privacy protection, and algorithmic ethics. Herington et al. argue that clinical use of AI-assisted diagnosis requires explicit patient awareness of AI’s role in their care decisions, proposing multidimensional oversight combining technical review, ethical evaluation, and cultural adaptability analysis (83). Privacy concerns are particularly acute given AI’s reliance on vast patient datasets, prompting recommendations for regulatory alignment with data protection laws to ensure proper anonymization and secure data usage. These interdisciplinary perspectives provide a macro-level view of AI’s impact on medical ethics and legal systems, urging policymakers to develop dynamic regulatory frameworks that integrate risk assessment, data compliance, and software updates. Such frameworks should establish “technical validation-ethical evaluation-clinical verification” feedback loops, safeguarding patient rights while fostering innovation.

### 4.5 Standards

Emerging research has focused on developing scientific methodologies to enhance regulatory efficiency for AIMD. Meng et al. argue that while traditional medical devices rely on clinical trials for evaluation, different evaluation parameters deal with different scenarios. The absence of scenario-specific performance standards leads to inconsistent evaluations, and the disconnect between regulatory pathways and development practices may create technical compliance barriers (72). To address these issues, more complex indicators are required, such as the area under the Receiver Operating Characteristic (ROC) curve of the algorithm and feature importance analysis. This body of research advocates for a dynamic regulatory paradigm based on a statistical risk perspective, which fosters the mutual advancement of regulatory science and AI technology by developing specifications compatible with GMLP and establishing a hierarchical, adaptive performance evaluation system (71, 72). Technical investigations explore alternative validation methods, including synthetic data and phantom testing, to reduce clinical trial costs while examining algorithmic performance under edge cases. Concurrently, regulatory scientists are developing standardized benchmark datasets for independent third-party validation of AI models, thereby supporting evidence-based regulatory decisions (84). Wang et al. identify widespread deficiencies in industry practices regarding explainability management, data traceability, and dynamic risk control, emphasizing that refined regulatory standards could catalyze sector-wide improvements (85).

International standardization bodies (e.g., IEEE, ISO) are actively formulating quality and risk management standards for medical AI. More specifically, the IEC has become a major hub for these efforts. The technical committee TC 62 (Electrical equipment in medical practice) and its subcommittees are particularly active, with project teams (e.g., PT 63450 on AI-enabled medical device safety and PT 63521 on the AI lifecycle) and advisory groups like the Software Network and Artificial Intelligence Advisory Group (SNAIG) leading the development of new standards for AIMD applications. Academic debates center on adapting ISO/IEC 62304 for machine learning software lifecycles and augmenting ISO 14155 with AI-specific validation methodologies. These efforts collectively advance the emerging discipline of “AIMD regulatory science.” Xue et al. contribute a risk-stratified AI governance framework, offering actionable guidance for developing adaptive regulatory systems (86). To bridge theory and practice, researchers emphasize tripartite collaboration among regulators, academia, and industry. Exemplary initiatives include FDA’s Digital Health Center workshops on AI validation and real-world performance monitoring, and MFDS’s leadership in promoting international regulatory harmonization through IMDRF (87, 88). Liu et al. conducted a systematic survey of 32 representative AIMD enterprises, employing descriptive statistical analysis to identify systemic quality management deficiencies. Their findings provide both targeted improvement recommendations for manufacturers and evidence-based support for regulatory standard development (85).

Collectively, academic research has established a multidimensional analytical framework for AIMD regulation, spanning macro-level policy comparisons to micro-level technical specifications. These contributions furnish critical evidence and innovative perspectives for the continuous refinement of regulatory policies.

## 5 The limitations and challenges of policies

Although major countries and regions have established regulatory frameworks for AIMD, these frameworks face significant challenges, many of which are now central topics in the academic literature, as discussed in the preceding section. The current system still has some limitations and difficulties in the face of the unique challenges of this emerging technology.

### 5.1 Post-market surveillance challenges for adaptive algorithms

Traditional medical device regulations assume that the algorithms of products are basically fixed after they are launched on the market, but AI software with self-learning capabilities breaks this assumption. If an AI diagnostic software continues to update its model parameters by learning new case data after its launch, its performance may gradually improve, but unforeseen changes may also occur. Most of the current regulatory systems have not yet fully covered this kind of “continuously evolving” product. Regulatory authorities often choose to require enterprises to lock the algorithm when going public. For instance, both China’s NMPA and Japan’s PMDA tend to approve the version of the “locking” algorithm (89). Although this ensures the certainty of the products on the market, it also weakens the capabilities and advantages of AI technology in an intangible way. Meanwhile, if the manufacturer does have significant improvements and needs to reapply, the process is lengthy and not conducive to timely correcting algorithm defects or deviations. Although the FDA’s PCCP and Japan’s PACMP offer a solution, that is, to pre-approve change plans, these mechanisms are still in their infancy and there are still urgent problems to be solved, such as the difficulty in determining the scope of algorithm changes, assessing the risks of algorithm changes, and verifying continuous learning algorithms. At present, how to allow the dynamic evolution of AI algorithms under the premise of security and controllability remains a prominent problem faced by regulators (34).

### 5.2 Challenges in clinical evaluation and efficacy demonstration

The performance of AIMD is often measured by indicators such as accuracy rate, sensitivity and AUC. Although these indicators can reflect the algorithm’s ability to detect diseases, they do not necessarily directly equate to clinical benefits. For instance, an imaging AI detects more tiny lesions, but does it truly improve the prognosis of patients? There is a complex causal chain. However, the current approval focuses more on technical performance and has limited coverage of clinical effects. Therefore, professional institutions and regulatory systems are putting forward corresponding management suggestions. In the 510(k) of the United States, many AI software are approved only based on retrospective data and there is no evidence of prospective randomized controlled trials. This is undoubtedly related to ethics and implementation difficulties, but it also means that some products lack rigorous evidence to verify their long-term safety and effectiveness when they are launched on the market. There are also limitations in risk capture such as adverse event monitoring and regular reporting after listing. Especially when the software has no hardware carrier and is widely distributed, the adverse event reporting system of traditional devices is difficult to detect the decline in algorithm performance or incorrect patterns in a timely manner. Therefore, some comments suggest that the current regulatory system may underestimate the actual risks of certain AIMD. This is a limitation that regulators must face up to and needs to be compensated for by strengthening post-listing research and evidence collection. For instance, enterprises are required to conduct real-world performance research within a certain period after going public and submit the results to the regulatory authorities for review (90). However, as of now, except for a few countries attempting real-world data as a supplement, such as the real-world pilot in Hainan, China, there is still a general lack of a systematic post-market evaluation mechanism for AIMD globally (91).

### 5.3 Algorithmic Bias and uncertain applicability

The decision-making quality of AI algorithms depends on the training data. If the training data cannot fully represent the target population, the performance of the algorithm on certain minority groups, such as minority races and patients with special diseases, may be poor. Hasanzadeh et al. believe that this is caused by non-representative data, bias in the reflection of basic data, etc. (89). For example, if an AI for skin diseases is mainly trained with skin images of European races, its accuracy rate may drop sharply when applied to patients in East Asia. The same problem may also be reflected in the differences in medical environments. For example, the applicability of data training from large hospitals to patients in primary hospitals is questionable. This bias is currently difficult to be fully detected through the limited pre-market verification. Regulatory requirements usually generally refer to “sample representativeness,” but lack clear standards or tests to guarantee it. In the approval process of various countries, the submitted datasets are mostly described in terms of quantity and basic characteristics, and there are few requirements for hierarchical performance such as race, age, and disease severity. This may lead to the approval and marketing of some bias algorithms. Bias not only affects fairness and effectiveness, but may also cause security risks, such as systematic misjudgment of certain groups. Furthermore, AIMD usually has specific intended uses, but in reality, doctors may apply it in situations beyond the scope. If the product manual does not clearly define the restrictions or the hospital lacks control, the algorithm may cause errors when used in unsuitable populations. Therefore, how to prevent and correct algorithmic bias in supervision and ensure that products are used within the indicated range is one of the weak links in the current policy. In the future, stricter data diversity requirements and usage supervision measures will be needed.

### 5.4 Model transparency and explainability

Complex models such as deep learning often have difficulty explaining their decision-making basis to users, which is called a “black box.” This brings troubles to both regulation and clinical use. When regulatory auditors evaluate such products, due to the lack of explainable information, it is difficult for them to fully understand the algorithm behavior merely based on the code and test results. This increases the uncertainty of approval. At present, regulatory agencies mainly rely on performance tests and development process documents to infer the reliability of algorithms, and have no understanding of their internal decision-making logic. However, in some high-risk applications, since the conclusion derivation process of the algorithm cannot be known, if AIMD gives results contrary to the doctor’s diagnosis, it may be difficult for doctors and patients to trust the algorithm. From the perspective of responsibility determination, when a black box algorithm makes a misdiagnosis, there is no way to analyze the source of the error (92). The AI Act of the European Union is considering requiring high-risk AI to have a certain degree of interpretability, which may in turn affect the technical design of medical AI products. Current medical device regulations, such as MDR, do not mandate that AI models be explainable, but some experts have suggested that necessary transparency requirements be included in regulatory guidelines. In the foreseeable future, insufficient transparency will become a significant limitation of the current policy. If not improved, it may weaken the trust foundation for the clinical promotion of AIMD.

### 5.5 Cross-domain complexity

AIMD integrates fields such as medicine, computer science, and data science, and regulatory agencies are confronted with challenges in terms of personnel and knowledge reserves. Many regulatory auditors have a background in biomedical engineering or clinical medicine and are not familiar with the principles of machine learning models, programming codes, etc. This leads to the fact that during the review process, the algorithm part mainly relies on the materials submitted by enterprises and lacks independent judgment ability. Some experts point out that the talents and tools in the current regulatory system are not yet fully ready to embrace the AI wave. Agencies such as the FDA have been recruiting data science talents and training existing personnel. However, overall, global regulatory agencies are still relatively weak in terms of specialized AI talents and technical means, which will limit the depth of policy implementation. Even if the regulatory requirements are complete, if the reviewers cannot effectively assess the quality of the algorithm, the regulatory effect will be discounted. The same issue also exists in the post-market stage, such as how to monitor an AIMD that is deployed in the cloud and constantly updated? The traditional methods of on-site inspection and document review are no longer fully applicable. It can be said that the lag in regulatory capacity building is a hidden danger of the current system, which requires time and resources to make up for.

### 5.6 Insufficient international collaboration

Globally, AIMD regulation shares commonalities, but currently, policies in various countries are fragmented. Enterprises need to obtain separate certifications in different markets, which increases both time and cost. Although organizations such as IMDRF are committed to coordination and unification, for instance, they have issued unified term definitions and are currently discussing GMLP guidelines (93), these condensations have not yet been elevated to regulatory requirements in various countries. Enterprises still need to meet different regulations one by one. Some scholars and industry insiders have called for strengthening the pilot program of mutual recognition of supervision or joint review to reduce repetitive work. However, due to the different legal systems and regulatory focuses of various countries, it is difficult to achieve a high level of mutual recognition in the short term. The insufficiency of international collaboration, to some extent, is also due to policy limitations, which makes the globalization of AI medical innovation not smooth enough. In addition, different countries have varying restrictions on cross-border data flows, which also affects the sharing of data from global multi-center clinical trials and algorithm training, and is not conducive to a comprehensive assessment of the performance of AIMD. These problems have not yet been solved in the current policies and need to be gradually overcome through inter-governmental cooperation and policy innovation (94).

## 6 Future trends

### 6.1 Promote a more flexible regulatory framework

Experts generally believe that future regulatory policies must break through the traditional rigid model and introduce adaptive and iterative dynamic regulatory concepts. The FDA’s Center for Digital Health has emphasized that AI software needs “Continuous Oversight.” Instead of managing the entire life cycle with a one-time approval approach, a dynamic oversight framework should be established through dynamic assessment studies of the product (95). On December 4th of 2024, the FDA finally determined the guideline titled “Recommendations for Market Submission of Intended Change Control Plans for Software Functions of AI Devices,” replacing the draft guideline released in April 2023. This means that algorithm updates have been incorporated into the FDA’s regulatory (14). Similarly, the PACMP is expected to verify the details in actual cases, thereby improving the execution process. Some experts even envision that in the future, AIMD might adopt a “rolling” approval system, where regulatory authorities regularly review the performance and improvements of algorithms and update product licenses based on the results, rather than having a one-time approval for a lifetime. This dynamic regulatory approach aligns with the rapid iteration characteristics of AI and is regarded as a mutually beneficial strategy for ensuring safety and promoting industry development. Of course, achieving such flexible supervision requires regulatory authorities to have strong data analysis and review capabilities. Therefore, corresponding resources need to be supplemented before promoting policies.

### 6.2 Emphasize the post-market supervision

Future policies will emphasis on post-market monitoring and management for AIMD. Agencies such as the FDA have stated that they will strengthen the real-world performance supervision of AIMD, for instance, requiring manufacturers to establish post-market monitoring plans and submit performance reports regularly. In the FDA’s AI action plan for 2021, it was mentioned to explore the use of real-world data and monitoring tools to promptly identify safety issues such as performance drift of AI products, so as to facilitate rapid intervention. Based on this, the FDA, Health Canada, and MHRA jointly established five guiding principles for the PCCP (96). EU experts also suggest that MDR should formulate specific post-market supervision guidelines for AIMD, such as updating technical documents more frequently and regularly reviewing training data. Due to the successful application of AI in-vitro diagnostic technology during the COVID-19, the MFDS has recognized the importance of real-time monitoring and rapid adjustment, and is considering establishing a national remote monitoring platform for medical AI products (97). It can be expected that in the coming years, major regulatory authorities may introduce post-market management requirements for AIMD, such as submitting annual reports, collecting user feedback, and re-training mechanisms. Meanwhile, real-world evidence (RWE) will play a more significant role in regulatory decision-making to make up for the insufficiency of evidence from randomized trials. This agile post-market supervision model is under discussion and is regarded as a key means to ensure patient safety in the AI era (98).

### 6.3 Employing “regulatory technology” for enhanced oversight

Interestingly, experts proposed that AI could be used to regulate AI. The regulatory authorities themselves are confronted with massive amounts of data and complex issues, and the efficiency of traditional manual review is limited. In the future, regulatory authorities may develop or adopt AI tools to screen declaration materials and detect risk signals. A tangible example of this trend is the emergence of open-source tools designed to assist with regulatory compliance, such as the COMPL-AI project, which provides a framework for checking generative AI models against the requirements of the EU AI Act (99); The FDA is developing applications that can automatically read and analyze source code to discover potential defects; European regulatory technology companies are also exploring the training of AI models to predict risk levels and assist regulatory authorities in determining the depth of review. Heinz et al. believe that regulatory technology will play a role in medical AI supervision, improving the coverage and accuracy of supervision through automation and intelligence (62). Of course, they also remind that excessive reliance on AI for decision-making should be avoided. The combination of humans and machines is the ideal model. In any case, as policies evolve, the informatization and intelligence construction of regulatory authorities themselves will be put on the agenda to match the complexity of AIMD.

### 6.4 Promote global standard alignment

As AIMD is a global focus, coordination at the international level will be further strengthened. For instance, the Key Term Definition Document of IMDRF was released in 2022, and the Principles of Good Machine Learning Practices entered the public comment stage in 2023 (53, 93). Experts predict that IMDRF may next initiate the formulation of guidelines related to the clinical evaluation and change management of AIMD to form a basic consensus. The World Health Organization (WHO) has also expressed its intention to develop a classification standard for digital health products, aiming to assist regions with insufficient regulatory capacity in adopting a unified standard to manage AIMD. The work of international bodies like the IEC’s TC 62 and its joint working groups on AI-enabled health informatics will be fundamental to creating these globally accepted technical requirements. The formulation of the EU AI Act has also indirectly prompted countries to consider an AI governance framework, which is expected to facilitate cooperation across jurisdictions. Industry experts from MFDS have called on regulatory authorities to actively participate in international dialogues and promote the coordination and alignment of regulatory requirements. For instance, it is possible to explore the establishment of a medical AI coordination mechanism similar to the International Technical Coordination Committee for the Registration of Pharmaceuticals for Human Use, so as to gradually converge global technical requirements and ultimately achieve one-time approval in multiple regions. Although it is difficult to achieve complete unification at the legal level in the short term, experts advocate starting with guiding principles and standards, allowing enterprises to prepare documents in accordance with unified norms, and referring to the same set of technical standards when countries conduct reviews. This will significantly reduce compliance costs and promote the global accessibility of AIMD. Countries such as South Korea and Canada have publicly expressed their support for the international unified guidelines, and the initial results may emerge in the coming years.

### 6.5 Pay attention to ethical and social impacts

Future regulatory policies will also incorporate more ethical considerations to ensure that AIMD is not only safe and effective but also patient-centered. Experts point out that if AI is not regulated, it may lead to problems such as intensified prejudice and privacy infringement, and erode the public’s confidence in medical AI in the long term. Therefore, some countries have begun to embed ethical principles in their regulatory frameworks. For instance, the AI Act of the EU explicitly stipulates that AI systems must adhere to requirements such as reliability, transparency, and fairness. These principles are expected to be concretized in the regulation of medical devices. Institutions such as the FDA have also emphasized the fairness and transparency of algorithms and funding research projects to develop methods for reducing bias (73). It can be foreseen that regulators may require enterprises to submit reports on the ethical impact of algorithms in the future to evaluate the effectiveness and fairness of products among different groups of people, as well as the anti-bias measures taken. Patient privacy protection will also be more prominently reflected in the approval process, especially for sensitive health data, to ensure compliance with strict privacy laws. Overall, “people-oriented” will become the guiding ideology for future AI medical regulation. Besides technical indicators, regulation will pay more attention to factors such as patient rights protection and doctor-patient relationships. Only AIMD that meets the standards in terms of safety, effectiveness and ethics can truly win the dual recognition of society and regulation (100).

### 6.6 Establish regulatory framework for emerging technologies

AI technology itself is constantly evolving. The emergence of new technology will bring about new regulatory issues, which require forward-looking planning. A prominent recent example is the application prospects of generative AI, such as ChatGPT. If a chatbot is used to provide health consultations or even diagnostic advice, does it fall under the category of medical devices? How can regulation ensure that such harmful suggestions are not produced? The Guidelines for the Approval and Review of Generative AIMD, which were first released by the MFDS in 2025, are responding to this trend (101). The FDA is also actively addressing this area. In late 2024, its Digital Health Advisory Committee released an executive summary on the ‘Total Product Lifecycle Considerations for Generative AI-Enabled Devices, ‘signaling a deep engagement with the unique challenges posed by these technologies, including issues of data quality, model transparency, and monitoring for emergent behaviors. While South Korea was the first to issue formal guidelines, the FDA’s detailed considerations demonstrate a move toward establishing a comprehensive U.S. framework (102). Experts predict that similar regulatory discussions will become increasingly frequent in the future. For instance, how will new technologies such as reinforcement learning and federated learning affect regulatory requirements? The larger the scale of an AI model and the more scattered its training data are, the more we should review its training process. All of these require the regulatory framework to be constantly updated. To this end, experts suggest that regulatory authorities maintain close ties with the academic and industrial sectors, establish a “regulatory sandbox,” allow new technologies to be piloted in a controlled environment and subject to regulatory observation, and then formulate corresponding regulations after summarizing experiences (103). The UK and Singapore have already implemented medical AI sandbox programs, and other countries may follow suit. Such cutting-edge explorations will pave the way for policies in advance and prevent regulation from lagging too far behind technology (104).

These anticipated shifts are directly informed by the limitations identified by regulators and the solutions proposed within the academic research community.

## 7 Conclusion

This study systematically reviewed the regulatory policies and academic research progress of AIMD in major countries worldwide from 2015 to 2025. The analysis revealed distinct regulatory frameworks among the U.S., the European Union, China, Japan, and South Korea. The U.S. employs flexible guidelines to accelerate innovation, while the European Union relies on stringent regulations to ensure safety. China drives domestic technological development through policy incentives, whereas Japan and South Korea focus on balancing robustness and efficiency. Despite these differences, all countries emphasize risk-based classification, full life-cycle management, and clinical validation. However, adaptive algorithm updates, data bias, and model transparency remain global challenges. Future directions should include the construction of dynamic regulatory frameworks, enhanced post-market surveillance, increased international collaboration, and stronger emphasis on ethical requirements, with regulatory frameworks for new technologies such as generative AI being laid out. The study concludes that regulatory policies need to strike a balance between safety and innovation, and rely on transnational coordination mechanisms to bridge standard discrepancies, thereby promoting the sustainable development of AIMD.
